# Iodine in Scrofula, Phthisis, Pulmonalis, and All That Class of Diseases, Which Depend on an Hereditary Tendency or Constitutional Taint

**Published:** 1857-06

**Authors:** B. Woodward

**Affiliations:** Galesburg, Ill.


ARTICLE II.—*Iodine in Scrofula, Phthisis, Pulmonalis, and all that class of diseases, which depend on an hereditary tendency or constitutional taint.* By B. WOODWARD, M.D., Galesburg, Ill.

That there is such a condition as that of 'taint' cannot be denied. That tubercular disease of every grade, including that form of it known as scrofula, depends on this taint or animal poison, is a proposition which few will be willing to controvert. That iodine, so far as experiments have been made with it, has proved an antidote for animal poisons none can deny.

The brilliant experiments of Prof. D. Brainard, with the woorara, and other animal poisons, and a few experiments made by myself, including a case reported by me in your journal for Feb., 1856, have shewn that it will neutralise the most virulent poison. Its use in the U. S. Marine Hospital, in Chicago, by Prof. Brainard, in the form of vapor, for the treatment of the most inveterate forms of ulceration, and its constitutional use, both in that Hospital, and in the Mercy Hospital, under Prof. N. S. Davis, in tubercular diseases, convinced all who had the privilege of following those hospitals the past winter, that it was an agent of great remedial power. The *modus operandi* of this medicine has been thought to be by promoting absorption; but, that this is only a secondary action seems to me clear. Its primary action seems to be by destroying the poison itself. 'Headland' in his invaluable work, on the 'action of medicines,' says: 'Iodine is not like mercury, a general antiphlogistic, but it is a stimulator of the function of absorption, as are all medicines that tend to impoverish the blood. Such medicines, when their action is pushed to an extreme, may, by destroying the albumen and fibrin of the blood, cause it to repair for its renovation, by means of the absorbent system, to the very tissues themselves. This action in causing absorption, can be no explanation of the blood-operations for which iodine is employed; for these are peculiar to it alone; whereas the other property is shared by other remedies. But this absorbefaciant action has doubtless been much exaggerated. By its action in neutralizing poisons in the blood, it is able to disperse any tumors which have a scrofulous or syphilitic origin.'

Adopting Headland's view, which I think to be the only correct one, we have the action of iodine in the diseases under consideration fully explained. It acts as a catalytic. It finds in the blood certain ingredients which do not exist there in a state of health, and its tendency is to destroy the morbid influences, and thus restore the system to a condition where the iron, the cod liver oil, and the glycerine, and other restoratives can come in and build up the wasted system. In that most dreaded disease, taberculous meningitis, its effects have been very satisfactory. The almost identity which exists between scrofula and true taberculosis, points most unmistakably to the same class of remedies in their treatment. I am strongly led to the belief that they are only different developments of one and the same disease. It is frequently observed that in families in which there is an hereditary tendency to scrofula, phthisis develops itself at an early age, and particularly are the children of such families subject to tuberculous meningitis. I have two such families now in my mind, in which this condition was strongly marked. Two of the children in one family, and one in the other, had died of what was called hydrocephalus. In both these families the other children had the emaciated bodies and limbs, and enlarged heads, which mark the tubercular diathesis. They were taken under a rigid hygienic treatment, coupled with the use of the iodide of iron, iodide of potassa and sarsaparilla, and in a few months I had the pleasure of seeing them strong healthy children. In the treatment of these diseases, remedy after remedy has been tried, and in its turn discarded from its not fulfilling the expectations of the profession; but there still remain a few which bid fair to answer our expectations: these are, cod liver oil, iodine, and its chemical combinations and glycerine.

The cod liver oil is undoubtedly one of our most reliable remedies in consumption, especially when combined with some of the iodides; but its tendency to disagree with delicate stomachs, makes its use impracticable in a large number of cases. In these cases the glycerine is fully indicated, and I think I have seen better effects from it when used as prescribed by Prof. N. S. Davis, in combination with the syrup of iodide of iron, than from the cod oil. Inhalations of iodine, combined sometimes with chloroform, and sometimes with conium, bring the remedy in immediate contact with the diseased lungs, and give relief from the distressing dyspnoea, which is so common a concomitant of tubercular disease of the lungs.

The profession have now, not only the means of forming a correct diagnosis in long diseases, by the means of the stethescope and percussion, but also of successfully treating, at least in their earlier stages, these most dreaded diseases. The day has passed by when 'consumptives' were restricted to the house, and a starving, debilitating treatment insisted on; we now urge, out of door exercise, plenty of fresh air, and a generous diet, with the use of such remedies as shall restore and invigorate the blood, and thus give tonicity to the system. Perhaps it is as much the fault of the profession itself, as of the people, that the treatment of lung diseases is so much in the hands of the quacks. Carelessness in making examinations of the chest has much to do with it. Bronchitis and taberculosis are too often confounded; want of patience in the treatment, is also another cause; and it may be that an imperfect knowledge, or erroneous opinion of the action of medicines is still another cause. Tubercular diseases are as much subject to the action of medicines as any others; and the medical profession suffer their patients to fall into the hands of the brotherhood of quacks, and their own time honored profession to be disgraced, by their carelessness. There are very many in the profession who are turning their attention to these diseases, but it must be acknowledged that a large proportion are folding their hands, and resting contented with 'the good old ways.'

